# Bilateral Dual Level Cervical Sympathetic Blocks to Treat Post-TBI Sequelae: A Case Report

**DOI:** 10.1155/crnm/2201504

**Published:** 2025-09-21

**Authors:** Michael J. Louwers

**Affiliations:** Reset Medical and Wellness Center, Strongsville, Ohio, USA

## Abstract

This study reports the clinical response and potential mechanisms of bilateral dual cervical sympathetic blocks, commonly referred to as stellate ganglion blocks (SGBs), in treating long-term symptoms following a mild traumatic brain injury (TBI). While previous research has shown that SGB can alleviate symptoms in patients with both TBI and posttraumatic stress disorder (PTSD), its utility for isolated post-TBI symptoms without concurrent PTSD remains unclear. In this case, a patient suffering from persistent symptoms for over a year following a mild TBI was successfully treated, suggesting that SGB may offer a viable and minimally invasive treatment option for individuals experiencing chronic post-TBI symptoms, even in the absence of PTSD.

## 1. Introduction

Mild traumatic brain injuries (TBIs) are the most common type of TBI, making up 75% of the cases [1]. They can result in persistent symptoms like cognitive, somatic, and emotional changes. In DSM-5, persistent symptoms after a mild TBI may be classified under neurocognitive disorder due to TBI (NCD-TBI) if there is an objective decline after the injury [2–4]. Common symptoms include headache, dizziness, nausea, sensitivity to noise, poor concentration, anxiety, fatigue, and irritability, which can decrease productivity and lead to financial strain [3–5]. Treatments include pharmacotherapy, physical rehabilitation, cognitive behavioral therapy, and activity modification [4, 5].

The stellate ganglion block (SGB), more aptly termed cervical sympathetic chain block (CSB), has long been used for pain management [6]. Successful treatment of anxiety disorders and posttraumatic stress disorder (PTSD) with CSB has been established in the literature [7–11]. A bilateral approach at C4 and C6 has been suggested as a preferred method for optimizing outcomes [11–13]. Mulvaney demonstrated in a retrospective analysis of patients treated for PTSD that underlying post-TBI symptoms improved after the procedure [14]. The research revealed a 55.9% improvement in symptoms at 1 week and a 53% improvement at 4 weeks, as measured by the Neurobehavioral Symptom Inventory (NSI) [14], which is a validated measure of post-TBI symptom burden [15, 16]. Patients in that study received treatment for PTSD as the primary indication [14], without acknowledgment of a formal TBI diagnosis.

Although PTSD and post-TBI symptoms overlap, CSB has not been proven to manage isolated post-TBI symptoms in patients without pre-existing PTSD. Mulvaney et al. acknowledged the overlap of symptoms and the lack of a formal TBI diagnosis as limitations of his study [14]. This case validates the benefits of CSB for isolated chronic post-TBI sequelae.

## 2. Case Report

The patient, a 22-year-old male with well-managed anxiety on duloxetine, suffered a mild TBI after hitting his head on a cement overhang in a parking garage. The next day, he visited urgent care for persistent headaches and dizziness. Examination and imaging showed no abnormalities, resulting in his discharge without treatment. Shortly after, he began experiencing dizziness, eye pain, headaches, increased anxiety, depression, nightmares, disrupted sleep, brain fog, sensitivity to light, and sensitivity to sound. A neurology consultation, brain CT, and MRI returned unremarkable results, leading to a diagnosis of NCD-TBI. He did not have a clinical diagnosis of PTSD, nor did he feel the injury itself was traumatizing. Prescribed treatments included physical therapy, vestibular therapy, gabapentin, and atogepant for headaches, while duloxetine was replaced with 150 mg of venlafaxine and 1.5 mg of cariprazine for worsening anxiety. Symptoms persisted despite treatment.

One year after injury, he sought treatment at our center for chronic post-TBI symptoms. As before, his examination was unremarkable. The patient consented to a bilateral dual-level CSB at C4 and C6. Treatment began on the right side under ultrasound guidance, using 10 mL of 0.5% ropivacaine, followed by the left side the next day. Before treatment, he scored 18 on the generalized anxiety disorder-7 questionnaire (GAD-7) [17] and 24 on the patient health questionnaire-9 (PHQ-9) [18], indicating severe anxiety and depression. He also scored 34 out of 88 on the NSI. An average NSI score in a patient with chronic post-TBI symptoms is 33 [15, 16].

At one week, the patient's NSI score improved to 15 (56% improvement), and at four weeks, to 12 (65% improvement). He reported significant reductions in headaches and sensitivity to light and sound. His sleep improved, and dizziness and vertigo resolved. Anxiety and depressive symptoms returned to his preinjury baseline (4-week GAD-7 score was 7, and PHQ-9 was 5). Moderate persistent eye pain led to a referral for monitoring.

## 3. Discussion

NCD-TBI is diagnosed when symptoms extend beyond 2 weeks although symptoms sometimes persist for months to years [3–5]. The traditional belief held that post-TBI symptoms stemmed from a diffuse mechanical injury to nerve cells [1, 5]. Secondary effects, like chronic inflammation, appear early after TBI, activating proinflammatory cytokines and immune cells. These release free radicals and inflammatory mediators [1–3], contributing to post-TBI pathological changes and impairing cerebral blood circulation. Pre-existing conditions such as anxiety, PTSD, ADHD, and migraines may predispose individuals to post-TBI symptoms [1, 3]. Whether stemming from the initial trauma, secondary inflammatory processes, or the exacerbation of pre-existing mental health conditions, the long-term effects of TBI can be profoundly debilitating and often resistant to treatment.

The treatment options are limited, including restricting activity, delaying sports, and reducing stimulation [4, 5]. If symptoms persist, neuropsychological testing can be completed, with consideration of behavioral, speech, and vestibular therapy as needed [4, 5]. Medication can help manage symptoms, including pain, vertigo, or anxiety, but limited additional treatment options exist [4, 5].

The CSB has long been used to treat pain due to cerebral vasospasm and specific nerve injuries such as CRPS [6]. Due to the multisystem reach of the sympathetic nervous system, including neuroendocrine, vasomotor, and regulatory, several other health benefits have been published, including benefits for anxiety, PTSD, long COVID, and menopause symptoms [7, 9, 10, 19]. Blockage of the sympathetic nervous system also reduces the inflammatory cascade and immune response, which may contribute to the reduction of secondary injury post-TBI [20–22]. It has been observed that a SGB can suppress the expression of proinflammatory factors during early systemic inflammation induced by severe trauma [20, 23]. The block's improvement of cerebral blood flow may also be part of the mechanism of symptom control post-TBI [24, 25].

This case report suggests that using a bilateral dual level CSB may significantly reduce the severity of chronic post-TBI symptoms, regardless of the presence of PTSD, or possibly other underlying conditions. The mechanism of this benefit may relate to the improvement in regional cerebral blood circulation and the reduction of chronic inflammatory mediators [14, 20, 22, 23, 26]. The confirmatory diagnosis of NCD-TBI, as well as the lack of pre-existing PTSD, makes this case unique and very promising in the field of TBI management. Currently, there is a need for further studies on patients with isolated chronic post-TBI syndrome to elucidate the potential indications, timing, and long-term benefits of CSB.

## Data Availability

The data supporting this case presentation are not publicly available due to patient confidentiality. Deidentified data may be available from the corresponding author upon reasonable request and with appropriate safeguards to protect patient privacy.
